# Effects of biperiden and acute tryptophan depletion and their combination on verbal word memory and EEG

**DOI:** 10.1007/s00213-017-4549-1

**Published:** 2017-02-16

**Authors:** Laura G. J. M. Borghans, Arjan Blokland, Anke Sambeth

**Affiliations:** Faculty of Psychology and Neuroscience, Department of Neuropsychology and Psychopharmacology, Maastricht University, PO Box 616, 6200 MD Maastricht, The Netherlands

**Keywords:** Acute tryptophan depletion, Biperiden, Episodic memory, ERP, Verbal learning

## Abstract

**Background:**

Research on the neurobiological foundations of memory has shown that multiple neurotransmitters play an important role in memory processing. To study the interaction between neurotransmitters such as acetylcholine and serotonin, pharmacological models can be used. In this study, we tested the effects of the muscarinic M1 antagonist biperiden, acute tryptophan depletion (ATD), and the interaction between the two on episodic memory using the verbal learning task.

**Methods:**

The study was conducted according to a double-blind, placebo-controlled, four-way crossover design. Seventeen participants received biperiden (2.0 mg), ATD (SolugelP), a combination of both, or a placebo in counterbalanced order with a wash out of at least 7 days. A verbal learning task was performed while recording electroencephalography. The task consisted of an immediate and delayed recall as well as a recognition part.

**Results:**

Results revealed decreased scores on the delayed recall after biperiden and ATD separately but no significant interaction between the two. However, the event-related potential components P3b, N400, and P600 did show an interaction during encoding.

**Conclusion:**

These results indicate that both BIP and ATD impair episodic memory. However, an interaction between the serotonergic and cholinergic system on memory performance is not supported.

## Introduction

The most common age-related neurodegenerative disorder leading to dementia is Alzheimer’s disease (AD). One of the major symptoms of this disease is episodic memory impairment, with impaired learning and recollection of new events. According to the cholinergic hypothesis formulated by Bartus et al. (1982), memory and other cognitive problems arise from degeneration of the basal forebrain cholinergic neurons. Traditionally, the non-selective cholinergic antagonist scopolamine has been used as a pharmacological model to mimic episodic memory impairments as seen in dementia (Ebert and Kirch 1998).

However, it has been suggested that scopolamine is a non-selective muscarinic receptor antagonist and has widespread effects in the brain and the body. Consequently, it can be anticipated that scopolamine may be associated with non-specific effects on cognitive performance (Klinkenberg and Blokland 2010). Therefore, a more selective drug may be more preferable as a pharmacological model for memory deficits. Biperiden (BIP) is a selective muscarinic M1 antagonist used for the adjunctive treatment of Parkinson disease (i.e., Akathisia). Cholinergic M1 receptors are located mainly in the cerebral cortex and hippocampus and to a lesser extent in the body and may therefore have less peripheral side effects (Klinkenberg and Blokland 2010). For example, in rats, BIP impaired short-term memory without impairing motor function (Ebert and Kirch 1998). In humans, similar results have been found (Sambeth et al. 2015; Wezenberg et al. 2005).

Although most research has investigated the role of acetylcholine in AD, cognitive impairments in AD may not be limited to impaired cholinergic functioning. AD is associated with a decrease in different neurotransmitters, including serotonin and noradrenaline (Trillo et al. 2013). Moreover, these neurotransmitters have also been found to play a role in cognitive functions. For example, the acute lowering of the amino acid tryptophan (ATD) has been used as a model for low central serotonin functioning (Young et al. 1988). It has been established that ATD impairs episodic memory functioning in patients as well as in healthy subjects (Mendelsohn et al. 2009; Sambeth et al. 2007). Since 5-HT is also reduced in AD brains (Trillo et al. 2013), it could be assumed that ATD could also be considered as a pharmacological model to test the effects of novel cognition enhancing drugs (e.g., van Donkelaar et al. 2008).

It is well known that the cholinergic and serotonergic systems interact anatomically and neurochemically (Cassel and Jeltsch 1995; Jeltsch-David et al. 2008; Lehmann et al. 2000; Richter-Levin and Segal 1993; Steckler and Sahgal 1995). However, a previous human study showed that administration of the selective serotonin reuptake inhibitor (SSRI) citalopram in combination with the muscarinic antagonist BIP did not confirm this finding, since the BIP-induced impairment was not reversed after administration of citalopram (Sambeth et al. 2015). This finding does not support an interaction between acetylcholine and serotonin on memory. On the other hand, it could be argued that this study was not conclusive since citalopram alone had no effects on memory and that therefore no interaction effects could be observed.

The aim of the current study was to further examine the interaction between acetylcholine and serotonin. We tested the effects of BIP and ATD alone and in combination on episodic verbal memory performance. Since BIP and ATD clearly impair episodic memory performance in healthy volunteers, we hypothesized that the combination of both treatments would decrease the scores on a verbal learning task more than treatment with either BIP or ATD alone would. Although BIP is a relatively selective model to induce episodic memory impairments and ATD is a more general model, both models have shown to be effective deficit models. ATD has shown to produce selective effects on episodic memory without affecting attention (Mendelsohn et al. 2009). To our knowledge, ATD is the only model to lower central 5-HT levels and induce memory impairments in healthy humans. Although the exact nature of the effects of ATD on the molecular level may not entirely be understood (van Donkelaar et al. 2011), experimental studies suggest a relation with brain 5-HT (Crockett et al. 2012).

Next to the behavioral tests, electrophysiological measurements were done, because these have shown to be more sensitive to changes than behavior alone (Luck 2005). Of particular interest were the P3a and P600 components of the event-related potential (ERP). The P3a has traditionally been linked to novelty processing, a prerequisite for memory encoding and a process that is influenced by the cholinergic system (Rangel-Gomez and Meeter 2016). We expected BIP to impair the P3a component more than ATD. Related to the P600 component, we expected both BIP and ATD to reduce its amplitude, because this component is related to memory encoding and retrieval (Jackson and Snyder 2008; Olichney et al. 2011), a process that was hypothesized to be impaired in this study.

## Method

### Participants

Healthy volunteers were recruited from Maastricht University through poster advertisements. Participants were required to be aged between 18 and 35 years. We decided on a restricted age range because EEG and ERPs can change with age and can be differentially sensitive to cholinergic modulation (Bennett et al. 2004; Fjell and Walhovd 2004; Pekkonen et al. 2005). Participants were also required to have a body mass index of 18.5–30.0. Female subjects were tested in the follicular phase of the menstrual cycle. They underwent medical screening before testing, consisting of a medical questionnaire and physical examination.

Exclusion criteria were past or current psychiatric, neurological, cardiac, gastrointestinal, hematological, hepatic, pulmonary, or renal illness; pregnancy; lactation; and excessive alcohol consumption (intake of >20 glasses/week). Subjects using any medication other than oral contraceptives, having a first-degree relative with a current or past psychiatric disorder, and presence of other deficits that could be expected to influence performance were also excluded. Participants were also excluded when they smoked more than ten cigarettes per day. On test days, participants were not allowed to smoke.

All subjects gave a signed informed consent before inclusion and were financially rewarded for their participation. The study was approved by the Medical Ethics Committee of Maastricht University.

In total, 17 participants of which 10 were female completed the study (mean age 22.4 years (S.D. = 3.0, range 19–29)). Four additional participants were recruited but did not complete all test sessions. Three of them discontinued due to the disliking of the taste of the protein drink and one stopped after two test days due to nausea. These participants were excluded from the analyses.

### Treatment and study design

The study was a double-blind, placebo controlled, four-way crossover design. The order of treatments was balanced over four test days and separated by a wash out period of at least 7 days.

BIP (Laboratorio farmaceutico S.I.T., Mede, Italy) is a muscarinic M1 antagonist used for the treatment of Parkinson symptoms. Peak plasma concentrations are reached 1–2 h after a single dose administration followed by a rapid decline to 12% of the peak value 6 h after intake. Common side effects on the central nervous system are drowsiness, vertigo, headache, and dizziness. Peripheral side effects consist of blurred vision, dry mouth, mydriasis, impaired sweating, abdominal discomfort, and obstipation. In this study, a dose of 2 mg was used, a dose well within the range of the recommended doses for BIP. Furthermore, research has found that an oral dose of 2 mg impaired cognitive performance in healthy adults (McShane et al. 2006; Sambeth et al. 2015; Wezenberg et al. 2005).

The tryptophan depletion treatment consisted of a protein drink containing 100 g of gelatin powder without tryptophan (TRP−); the composition of the mixture was the same as the one used in previous studies in which cognition impairing effects were found (e.g., Evers et al. 2005). Previous studies in man have shown that this treatment consistently leads to a 70–75% decrease in plasma TRP levels (Evers et al. 2005; Stenbaek et al. 2016). The placebo treatment consisted of an identical protein drink to which 1.21 g tryptophan is added (TRP+). The protein drink was prepared by adding 200 ml of tap water to 100 g of gelatin powder (Solugel P, PB Gelatins, Belgium). Both drinks had an identical taste.

### Procedure

After inclusion in the study, the participants first performed a training session. During this session, all cognitive tests were practiced to familiarize the participants with the study procedures and minimize procedural learning effects.

Each test day started with the assessment of the general status, filling in questionnaires and taking a blood sample. This was followed by the intake of either the ATD or balanced drink. Three hours after the intake of the drink, the participants received the capsule containing either BIP or a placebo. During the waiting period, participants were placed in a room where they could study, read, use their laptop, or watch television.

After this, the participants were offered a low-TRP, low-protein lunch. Four hours after the intake of the drink, the experiment was started.

### Questionnaires

To assess subjective alertness, the Bond and Lader mood scale was used (Bond and Lader 1974). Adverse effects were assessed with a self-report questionnaire consisting of 31 possible complaints to be rated on a 4-point scale. Only dry mouth, sleepiness, nausea, headache, dizziness, fatigue, and drowsiness were analyzed.

### Verbal learning task

The used test is an adapted version of the Rey auditory verbal learning test (Lezak 1995), which assesses short- and long-term memory function for verbal information. The test consisted of a list of 30 monosyllabic words (18 nouns and 12 adjectives) in English, which were presented on a computer screen for 1 s. The words were presented three times in the same sequence, and immediately after presentation, a free recall phase followed (immediate recall; sum of words recalled in three trials). Thirty minutes after the third trial, the participant was asked to freely recall as many words as possible (delayed recall). Subsequently, a recognition test was presented, consisting of all former words and 30 new but comparable words (distracters). The words were shown on a computer screen for a max of 1500 ms, and participants were asked to rate whether they were presented in the learning trials by a “yes/no” response. A new trial started 3500 ms after presentation of the previous word.

Each session, a different word list was presented to the participants. The order of the lists was balanced across assessments. Outcome measures were the number of words correctly recalled in the three immediate recall trials and delayed recall phase. In the recognition test, mean reaction times were measured in milliseconds as well as the number of correct recognized words.

The main behavioral outcome measures were based on recall scores. In contrast, the ERP-related measures were extracted during encoding and recognition. The ERP components P3a, P3b, N400 and P600 were compared to examine whether the initial stimulus processing during the learning trials differs from word to word. Finally, ERPs for the old and new items during the recognition task were measured.

### EEG acquisition

An EEG cap was used to place a set of 32 EEG electrodes according to the international 10–20 system (Jasper 1958), but only the midline electrodes (Fz, FCz, Cz, CPz, Pz) were used in the statistical analysis. A reference and a ground electrode were placed at the linked mastoids and at the forehead, respectively. Eye movements were detected by horizontal and vertical electro-oculogram (EOG) recordings. Before electrode attachment, the positions were cleaned with alcohol and slightly scrubbed with a gel in order to provide good conductance. Both EEG and EOG were filtered between 0.01 and 100 Hz and sampled at 1000 Hz.

The EEG data was analyzed using Vision Analyzer 2 (Brain Products, Gilching, Germany) software. Epoch files were made from 100 ms before stimulus onset until 1000 ms after onset, using the last 100 ms before stimulus onset as baseline. High-pass (1 Hz) and low-pass (30 Hz) filters were applied offline. The segments were checked for EOG activity (visually and by using the Gratton and Coles method in Vision Analyzer) and other artifacts and excluded if an artifact occurred during the first 1000 ms after stimulus presentation. Next, averages were calculated for each stimulus type and treatment. The grand average was used to determine the ERP components. For the VLT, peak detection windows were defined as the most positive or negative value between the following intervals: P3a 210–290 ms, P3b 290–360 ms, N400 340–470 ms, and P600 450–700 ms. For the VRT, the windows were P3a 210–280 ms, P3b 280–350 ms, N400 310–460 ms, and P600 430–720 ms.

### Statistical analysis

In this study, a 2 × 2 × 5 factorial design was used to analyze the outcome variables using repeated measures multivariate analyses of variance (MANOVA). The two treatment conditions (ATD: ATD or placebo and BIP: BIP or placebo) and electrode positions were entered as within-subject factors. Finally, effect sizes (η^2^_partial_) were calculated to determine the magnitude of the effects (see Table 3). All statistical analyses were done by using the Statistical Package for the Social Sciences for Windows (version 24; SPSS Inc., Chicago, IL, USA).

Adverse effects and the Bond and Lader were also analyzed using ANOVA. For this analysis, differences between effects at test vs. at baseline were taken into account. Post hoc testing was performed with a least significant difference (LSD) test.

## Results

### Questionnaires

B&L alertness: No significant effects of BIP or ATD (F(1, 16) = 1.21, *p* = .288 and F(1, 16) = 2.755, *p* = .116, respectively). No interaction between the two treatments was found (F(1, 16) = .571, *p* = .461).

As for the adverse effects, participants reported a significant increase in drowsiness after biperiden as compared to placebo (F(1, 16) = 7.314, *p* = .016, ηp^2^ = .314). Neither of the other aspects was affected by any of the treatments, nor were interactions between biperiden and ATD found (see the most common adverse effects in Table 1).

**Table 1:** Mean difference scores as change from baseline (SE) for the outcome variables of the questionnaires

	Placebo	BIP	ATD	Combination
Bond and Lader alertness	−125.41(36.21)	−120.82 (35.67)	−53(34.39)	−107.12 (33.19)
Adverse effects				
Headache	0.47 (.21)	0.41 (.17)	0.24 (.20)	0.29 (.14)
Sleepiness	0.53 (0.29)	0.53 (0.21)	0.35 (0.28)	0.41 (0.19)
Dizziness	−0.06 (0.06)	0.06 (0.6)	−0.06 (0.10)	0.06 (0.10)
Nausea	0.18 (0.13)	0.24 (0.19)	0.0 (0.09)	0.18 (0.18)
Dry mouth	−0.12 (0.08)	−0.06 (0.16)	0.06 (0.18)	−0.06 (0.14)
Fatigue	0.47 (0.21)	0.65 (0.19)	0.24 (0.28)	0.41 (0.17)
Drowsiness	0.77 (0.20)	0.77 (0.22)	0.47 (0.24)	0.59 (0.17)

Negative numbers indicate a decrease and positive numbers indicate an increase in the feeling

### Behavioral results of VLT and VRT

**Fig. 1:**
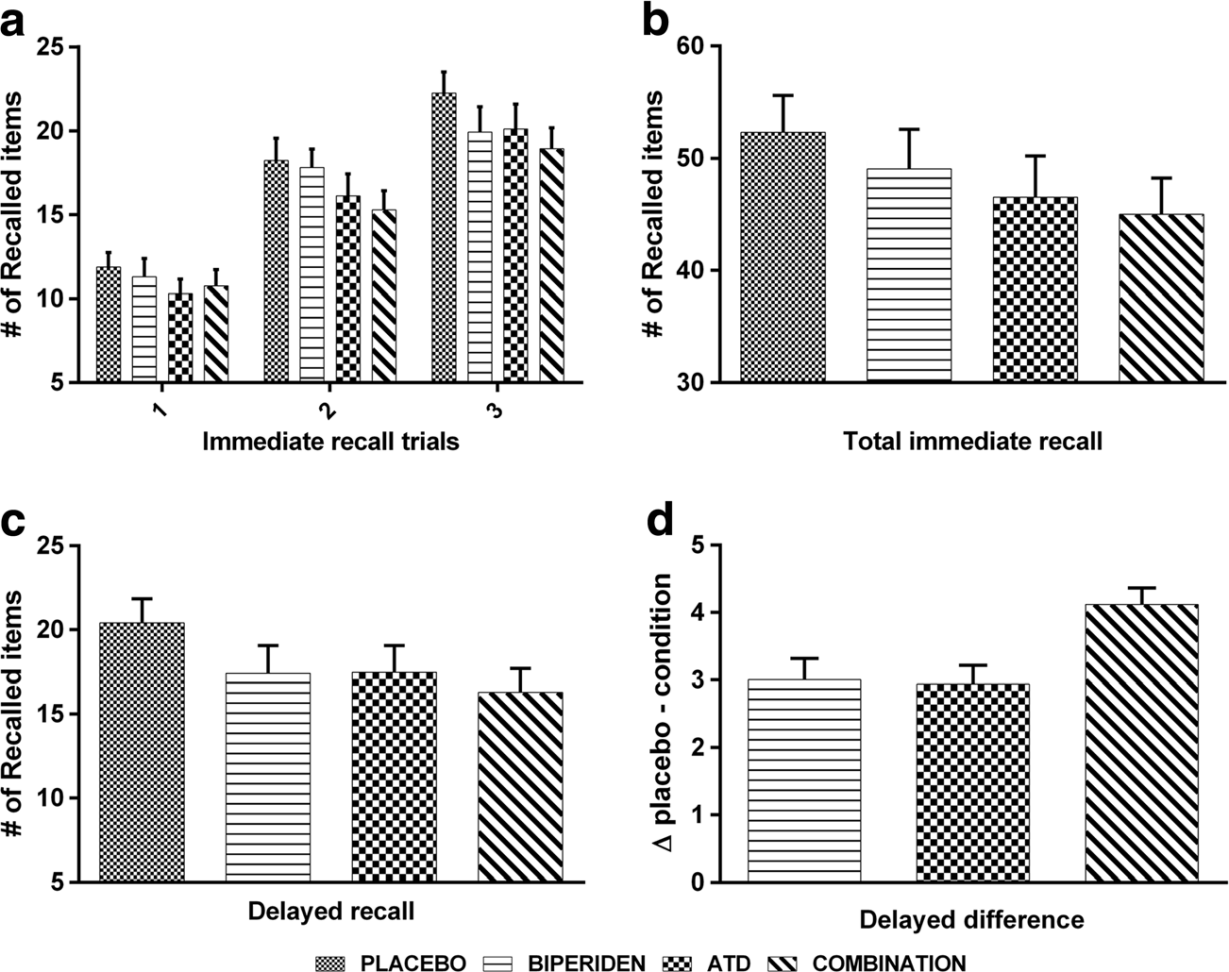
The number of words recalled during each immediate recall trial (**a**); the total number of words recalled during the three immediate recall trials (**b**); the number of words recalled during the delayed recall (**c**); placebo—condition difference scores for the delayed recall (**d**)

Figure 1 as well as Table 2 show the total immediate and the delayed recall scores during the VLT for all four treatments. ATD significantly impaired immediate recall (F(1, 16) = 9.04, *p* < .009, η_p_^2^ = 0.361). BIP, however, did not significantly impair immediate recall (F(1, 16) = 1.98, n.s.). Additionally, there was no interaction between the two treatments for immediate recall (F(1, 16) = .358, n.s.).

**Table 2:** Mean scores (SE) for the outcome variables of the verbal learning task and verbal recognition task

	Placebo	BIP	ATD	Combination
Verbal learning task (VLT)				
Total immediate recall	52.35 (3.24)	49.06 (3.53)	46.53 (3.45)*	45.00 (3.23)*
Delayed recall	20.41 (1.43)	17.41 (1.65)*	17.47 (1.58)*	16.29 (1.42)*
Verbal recognition task (VRT)				
Number of correctly recognized words	27.24 (0.49)	26.88 (0.75)	27.50 (0.64)	26.74 (0.69)
Mean reaction time for correct responses to old and new stimuli (in ms)	662 (13)	659 (13)	677 (17)	686 (22)

*p < 0.05 (of note, asterisks are shown for both BIP and combination or ATD and combination because they together reflect the main effect of the treatment)

As for the delayed recall, both ATD and BIP significantly impaired memory performance (F(1, 16) = 13.71, *p* < .003, η_p_^2^ = 0.461 and F(1, 16) = 5.61, *p* < .032, η_p_^2^ = 0.259, respectively). Again, no interaction between the two treatments was found (F(1, 16) = .885, n.s.; see Fig. 1c).

Furthermore, difference scores reveal no differences between treatment conditions (see Fig. 1d). Paired samples *t* test showed that there was no significant difference in the scores for BIP (M = 3.00, SD = 5.39) and ATD (M = 2.94, SD = 4.67); t(16) = −0.06, *p* = 0.957). Furthermore, the difference between ATD and COMBI (M = 4.12, SD = 4.18) was also not significant: t(16) = −0.90, *p* = 0.384. And, the difference between BIP and COMBI was not significant: t(16) = −1.09, *p* = 0.322.

In the recognition test, participants significantly recognized more new than old words correctly (F(1, 16) = 5.39, *p* < .035), but they responded faster to old than new words (F(1, 16) = 8.56, *p* < .011). Neither of the two treatments affected recognition performance and reaction time during the delayed recognition paradigm, nor did the treatments interact (all F values < 2.75), (see Table 2).

### EEG results of VLT

#### P3a

**Fig. 2:**
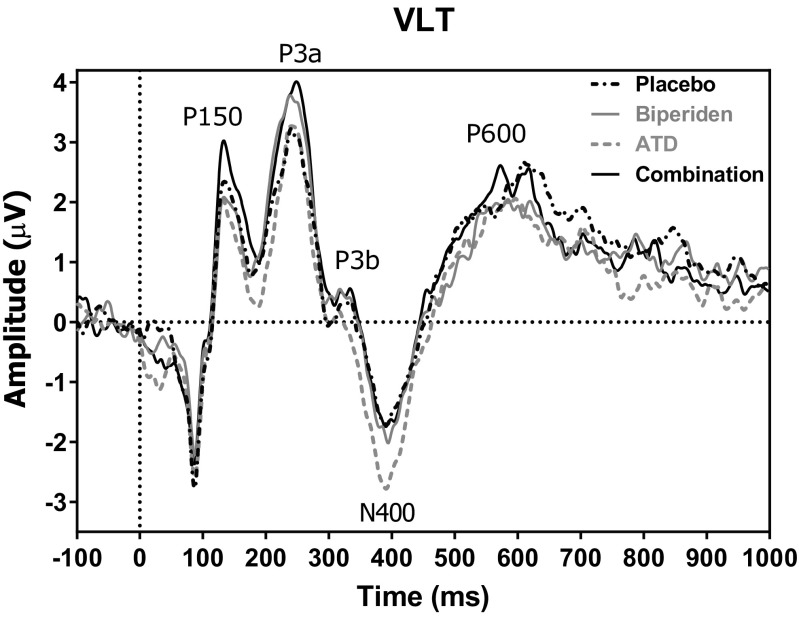
Grand average across participants of the ERPs elicited by the treatments at the Cz electrode

A main effect of BIP was found for the main analysis (F(1, 16) = 8.29, *p* < .012), (see Fig. 2 and Table 3). BIP significantly increased the P3a amplitude. ATD did not affect the P3a amplitude (all F values < 0.78).

**Table 3:** Mean amplitude in μV (SE)

VLT	Placebo	BIP	ATD	Combination		BIP	ATD	Interaction
P3a	4.9 (0.9)	5.7 (0.8)	4.8 (0.9)	5.4 (0.9)	F	8.29*	0.78	0.17
					η_p_^2^	0.341	0.046	0.011
P3b	2.8 (0.5)	2.9 (0.5)	1.9 (0.7)	3.1 (0.6)	F	3.41	0.66	5.26*
					η_p_^2^	0.176	0.040	0.247
N400	−2.8 (0.5)	−3.3 (0.4)	−3.9 (0.5)	−2.3 (0.5)	F	0.37	1.48	9.92*
					η_p_^2^	0.023	0.085	0.383
P600	4.9 (0.5)	4.2 (0.4)	4.1 (0.3)	4.5 (0.4)	F	0.57	0.65	7.67*
					η_p_^2^	0.034	0.039	0.324
VRT	Placebo	BIP	ATD	Combination		BIP	ATD	Interaction
P3a	3.7 (0.8)	4.7 (0.9)	3.4 (0.8)	5.0 (0.9)	F	12.94*	0.00	0.36
					η_p_^2^	0.447	0.00	0.022
P3b	1.7 (0.8)	2.5 (0.6)	2.10 (0.8)	2.8 (0.8)	F	3.83	1.92	0.04
					η_p_^2^	0.193	0.107	0.002
N400	−4.4 (0.7)	−3.6 (0.6)	−4.5 (0.8)	−3.4 (0.7)	F	7.05*	0.02	0.23
					η_p_^2^	0.306	0.001	0.014
P600	7.7 (0.8)	7.5 (0.8)	8.2 (0.7)	7.5 (0.6)	F	1.39	0.44	0.61
					η_p_^2^	0.080	0.027	0.036

For each effect, F values and effect sizes (ηp^2^) are reported

*p < 0.05

P3a latency was not affected by any of the treatments (all F values < 2.75).

#### P3b

A significant interaction between BIP and ATD was found in the main analysis (F(1, 16) = 5.26, *p* < .037). This effect was caused by the fact that ATD treatment reduced P3b amplitude as compared to the other treatments, indicating that BIP reversed this decrement.

None of the other measures related to any of the treatments in the main analysis for latency were significant (all F values < 2.50).

#### N400

A significant interaction between BIP and ATD was found for the main analysis (F(1, 16) = 9.92, *p* < .007). The N400 amplitude was more negative after BIP and after ATD treatment than after placebo. Furthermore, placebo and the combined treatment did not differ, indicating that the amplitude normalized after combined treatment.

#### P600

An interaction between BIP and ATD was found in the main analysis (F(1, 16) = 7.67, *p* < .015). The P600 was reduced after BIP and after ATD but only to a minor extent after the combined treatment.

Regarding latency, an interaction between ATD and electrode was found in the main analysis (F(4, 64) = 3.783, *p* < .021. Whereas the latencies were similar for all electrodes after Placebo, ATD reduced the latency at CPz and Pz.

Regarding latency, no interactions were found in the main analysis (all F values < 0.93).

### EEG results of VRT

#### P3a

A main effect of BIP was found for P3a amplitude (F(1, 16) = 12.94, *p* < .003), (see Fig. 2 and Table 3). P3a amplitude was increased after BIP as compared to placebo. No other effects related to any of the treatments were found regarding either the amplitude or the latency.

#### P3b

An interaction between BIP and ATD was found for the latency of the P3b (F(1, 16) = 7.81, *p* < .014). P3b latency was reduced both after ATD and after BIP as compared to placebo but was not different from placebo after the combined treatment.

An interaction between BIP and electrode was found (F(4, 64) = 7.746, *p* < .003). This was caused by the fact that the P3b amplitude for BIP was larger than for placebo at Fz and FCz, although this difference was not present for Cz, CPz, and Pz.

#### N400

A malin effect of BIP was found for N400 amplitude (F(1, 16) = 11.10, *p* < .005). Amplitude after BIP was less negative than after placebo. The ATD and BIP conditions separately did not differ from placebo.

BIP also significantly interacted with electrode (F(4, 64) = 6.725, *p* < .006). BIP was less negative than placebo at all electrodes, but this effect was most pronounced for the Fz, FCz, and Cz electrode positions.

With regard to N400 latency, a main effect of BIP was found (F(1, 16) = 6.32, *p* < .024). Latency was slightly increased after BIP.

#### P600

No significant effects related to any of the treatments were found for the amplitude of the P600 (all F values < 1.39).

## Discussion

The aim of this study was to examine the effects of BIP and ATD on episodic verbal memory and their electrophysiological correlates in healthy adults. Additionally, the interaction between the serotonergic and cholinergic system was examined by combining both treatments. BIP significantly decreased the scores for the delayed recall but not for the immediate recall. ATD resulted in lower scores for the immediate recall as well as for the delayed recall. However, the combination of BIP and ATD did not impair the memory performance more than for each treatment alone. Thus, no interactions between BIP and ATD were found. Analysis of the EEG data revealed that BIP increased the amplitude of the P3a component for both the acquisition phase and the recognition phase of the verbal learning task.

The current study again confirms that an M1 antagonist is effective in inducing memory impairment in a verbal word learning task. However, in our study, BIP only impaired delayed recall whereas in two other studies, immediate as well as delayed recall was impaired (Wezenberg et al. 2005; Sambeth et al. 2015). In all studies, similar doses and drug-test intervals were used. Of note, a more detailed analysis revealed that BIP impaired the performance at the third trial of the immediate recall (*p* < 0.05). Thus, there is some support that BIP also impaired immediate recall, although not as robust as in previous studies.

During the memory encoding phase and the recognition trials, a significant effect of BIP was found for the amplitude of the P3a component. The amplitude of this component was larger after BIP than after the other treatments. As mentioned previously, this component is involved in novelty processing; larger amplitudes correspond with stimuli that are perceived as more novel (Rangel-Gomez and Meeter 2016). This may relate to a poorer immediate recall performance after BIP treatment since the stimuli remain more novel even after three presentations.

According to the literature, the amplitude of the N400 decreases after repeated stimuli, thus indicating that stimuli are consolidated (Olichney et al. 2011). Surprisingly in this study, even though the N400 amplitude was as expected increased during encoding, the amplitude was less negative after BIP compared to the other treatments during the verbal recognition task. This suggests that in the BIP condition, words were consolidated better compared to the other condition. This is against our expectations and behavioral findings since we expected BIP to impair memory-related components. Possibly, the relatively large amplitude of the P3b component may have influenced the N400 amplitude in this condition. However, this probably does not fully explain the decreased N400 amplitude. Lastly, we hypothesized that the P600 amplitude would be decreased after BIP; however, we did not find any effects on both encoding and recognition. Altogether, EEG data suggest BIP mostly affects novelty processing, whereas other memory-related ERP components were not affected by BIP.

It is presumed that ATD impairs cognition by lowering central serotonin levels (Klaassen et al. 1999; but see van Donkelaar et al. 2011). According to a review (Mendelsohn et al. 2009), most studies using acute tryptophan depletion report impaired delayed recall but not immediate recall. Also, they concluded that ATD impairs the consolidation and to a lesser extent encoding of episodic memory. The present study corroborates these previous studies and further supports the notion that ATD has a robust effect on verbal memory performance. The impairment is consistently found on delayed recall, whereas the effects on immediate recall seem to depend on the sex of the subjects (Sambeth et al. 2007), larger impairments being found in females. In the current study, 10 of 17 subjects were female, which may explain the effect on immediate recall in our study.

Even though memory performance on both the immediate and delayed recall was decreased after ATD, the task-related ERPs were less affected by ATD. Unlike BIP, ATD did not affect the P3a component that reflects novelty processing. This could indicate that ATD and BIP both act on different processes in the brain. Apparently, ATD has a different effect on brain processing and 5-HT may not be involved in novelty processing (Rangel-Gomez and Meeter 2016). On the other hand, some genetic studies suggest a role of the 5-HT2 receptor (Schott et al. 2011) and the 5-HT transporter (Enge et al. 2011). These mixed findings on the role of 5-HT on novelty processing is likely to be related to the type of manipulation of the 5-HT system. The N400 was more negative after ATD as compared to placebo during the encoding phase. Given that it should decrease after repeated stimulation (Olichney et al. 2011), the increased N400 indicates that ATD likely impairs processes related to consolidation.

The objective of the current study was to examine whether there would be additive effects of BIP and ATD treatments (i.e., an interaction effect). We expected that the scores for the verbal learning task would be decreased more as compared to both the ATD and BIP alone conditions. In contrast to our hypothesis, the behavioral results did not reveal an additive effect of the combined ATD BIP treatment. No interaction effects were found for the behavioral data for the immediate and delayed recall or the recognition test. The ERP data resulted in mixed outcomes which are difficult to interpret in relation to the memory performance. Some interactions between BIP and ATD were found for the amplitudes of the P3b, N400, and P600 components on the encoding trials. However, the interpretation of this interaction is not easy since no interaction on memory performance was found. During the verbal recognition task, no interactions on amplitude were found at all.

The ATD method is based on the assumption that the depletion of tryptophan, a precursor of 5-HT, leads to decreased levels of 5-HT in the brain. Currently, this appears to be the most relevant model to affect central 5-HT functioning. However, the notion that ATD influences memory only by lowered central 5-HT levels was questioned recently (van Donkelaar et al. 2011). It was suggested that alternative pathways may also lead to memory impairments (e.g., LTP/NMDA related mechanisms). For example, ATD leads to lower kynurenic acid levels and has been associated with cognitive performance (Kennedy et al. 2015). However, there is no clear evidence that the changes in kynurenic acid levels affect the cholinergic neurotransmission (e.g., Stone and Darlington 2013). On the other hand, it has been claimed that the observed effects after ATD are closely related to 5-HT-related functions (Crockett et al. 2012). Although the exact nature of the molecular effects of ATD is not fully understood, the current study provides no support for an interaction between ATD (5-HT or NMDA-related) and BIP (ACh/M1 receptor) on memory performance. Further studies are needed to further examine whether this lack of interaction between neurotransmitter systems is related to NMDA or 5-HT.

Related to the discussion above, it should also be noted that BIP has a selective affinity for the M1 and M4 receptors (Perez et al. 2006), whereas ATD has a global effect on serotonin and kynurenine (Badawy and Dougherty 2016; Kennedy et al. 2015). Even though in this study a selective and a global challenge were combined, both methods are assumed to act on different neurotransmitter systems in the brain. Since both treatments impair memory performance, we expected a stronger impairment after a combined treatment. However, the present study did not find support for an additive interaction effect. On the other hand, this does not exclude that both systems could interact, as was shown with the EEG-related measures. Further studies could explore the interaction between acetylcholine and more specific serotonergic drugs (e.g., Wingen et al. 2007).

The lack of relation between EEG measures and behavioral measures could be explained by at least two factors. First, the ERPs were recorded during the encoding phase and the recognition phase, whereas the effects on memory performance were observed during the immediate and delayed recall test. Since these are independent processes of memory, the ERPs may not directly relate to the recall scores. Secondly, the effects of ATD and BIP could be related to processing in brain areas that were not detected by the scalp electrodes. For example, it has been found that the hippocampus is important for word learning (Shtyrov 2012).

The results for the treatment effects on the ERP components are more ambiguous compared to the behavioral data. Most effects were found related to the amplitude of the P3a component, implicating that novelty detection was affected. Related to BIP treatment, this indicates impaired encoding. Likewise, the P3a amplitude was increased for the recognition trials in the presence of BIP, indicating that impaired encoding is a longer lasting process that can last up until the delayed recall. The N400 was impaired after ATD, indicating that this manipulation rather leads to impaired consolidation. Significant interactions were found for the amplitude of the P3b, N400, and P600 during encoding. Altogether, we can conclude that the presence of BIP and ATD alone did impair episodic memory. However, against our expectations, an interaction between BIP and ATD was not found.

In conclusion, this study does not support the notion that the serotonergic and cholinergic systems interact in processes related to learning and memory on the behavioral level. On the electrophysiological level, there are indications that a possible interaction exists. Further research is needed to scrutinize the exact nature of the interaction between the cholinergic and serotonergic systems in relation to memory functions.
